# They Shoot Horses, Don’t They? A warning to medical schools about medical teacher burnout during COVID-19

**DOI:** 10.15694/mep.2021.000047.1

**Published:** 2021-02-16

**Authors:** Ken Masters

**Affiliations:** 1 Sultan Qaboos University

**Keywords:** Medical teacher burnout, COVID-19, medical education, emergency remote teaching, online learning, e-learning, technology

## Abstract

This article was migrated. The article was marked as recommended.

The 1969 film, *They Shoot Horses, Don’t They?* provides a contextual background for what appears to be happening to medical teachers as they attempt to cope with teaching during the COVID-19 pandemic, staving off the threat of exhaustion. This short piece argues that it is necessary for medical education institutions to recognise the changing demands made on, and by, their teachers, so that they can prevent burnout, and provide the support required to take online teaching to the levels that will now be expected. It traces the medical teachers’ changes across three stages of development, commenting on the overall mood, attitudes towards students, teaching focus, research focus, computer usage, theoretical knowledge and self-growth, assessment and institutional support. The aim is to provide some degree of insight into medical teachers’ needs so that adequate anticipation and responses can occur before it is too late.

## Introduction

The 1969 film, *They Shoot Horses, Don’t They?* ( Pollack, 1969) (based on Horace McCoy’s 1935 novel of the same name ( McCoy, 1935)) shows the desperation of people attempting to reach a mostly unreachable goal; not only are they unprepared for the demands of what is to come, but the situation is unpredictably altered by those in charge as the participants proceed. As a result, some lose hope and stop; some move on to better opportunities; most are knocked out by sheer exhaustion. The rest keep on going through the motions of what is expected of them.Yowza! Yowza! Yowza!

As a medical educator who has been researching and publishing in the field of online medical education for more than 15 years, I noted the important impact of the COVID-19 pandemic on medical education colleagues: the move to massive online teaching and learning, initially characterised by Emergency Remote Teaching (ERT) ( Hodges *et al.,* 2020). In particular, I noted those medical teachers who previously had little or no experience with online teaching, and who are now heavily engaged in it.

Some medical schools may have access to large support departments replete with instructional designers, but, given the fact that so many higher education institutions had to adopt ERT, this is likely to be a minority. Where this support does exist, they, themselves are overwhelmed with the demands ( Bessette, 2020). While the change to online learning was unquestionably necessary, it is important for medical schools to reflect upon what has happened over the past year, to think about what is to come, and to ensure that the impact on their teachers does not echo the film’s “epic of exhaustion” ( Canby, 1969).

## What to do?

Perhaps a starting point is for the institution to realise that online teaching is not merely a virtual version of what has always happened in the face-to-face class. Any decision-maker who comments to that effect will not only indicate a lack of awareness of the real situation, but will also cause a loss of confidence in the institution.

The second point is to realise that your teaching staff have also changed. The teachers are no longer the same people they were a year ago, and their needs and expectations have grown. Institution administrators need to recognise and respond to the new situation, or, if they cannot, then step aside and let somebody else do it.

## A guide to the changes

### Purpose of the guide

In order to assist in this understanding, this paper presents a simple guide ( Table 1) that indicates the progression of the teachers through three stages. It is written somewhat colloquially and in the first person, and the idea is for institutional managers to try to judge the current placing of many of their teachers, respond to current needs, and anticipate future needs.

Not everybody will progress in the same way and at the same speed, so no timeline on this progression exists. Either way, one would imagine that most medical teachers will be able to see themselves somewhere in this table.

### Clinical Relevance

It should also be noted that Table 1 has a focus on the teaching activities, and does not address the corresponding developments in clinical health care delivery that face many medical educators. This does not mean that clinical teachers are ignored; on the contrary, given that COVID-19 has, directly or indirectly, substantially complicated the provision of healthcare, most clinical teachers are doubly-burdened, and so require an even greater and targeted response from the education institution.

**Table 1:** Characteristics of stages through which medical teachers have moved, and may move, because of the pandemic

Stage / Charact.	1: Emergency flurry	2: Settling into a dusty road	3: The long haul
**Overall Mood**	Fear, trepidation, enthusiasm, excitement.	A little calmer, but there is now pressure to deliver better quality teaching. Fear that I cannot keep the pressure at this level.	Reflection, interspersed with panic, long-term planning, fighting the encroaching exhaustion. Is everyone this tired? (Can I get fed by tube?)
**Attitudes towards students**	We’re all in this together. We know you are struggling, so are we. Please have patience, we’re doing what we can. This won’t last, we will get through this together. (And they ask me for advice on how to study under these circumstances? How should I know? I’ve never had to do this. All I can do is trot out platitudes and clichés, and try to calm them.)	This is continuing. How can we make this better? How can I be available for my students who seem to need me 24/7, without compromising family life and my own sanity? We have come a long way, and we are coping...I think. But how are my students coping? How often should I check in with them to see how they are doing? I cannot assume their tech familiarity. How will this affect students’ evaluations of my course? Should I care? Should I adjust my teaching to get good ratings?	This is longer than we anticipated, but please see it as part of your professional training; this is how you will be dealing with patients and colleagues. But all the time I am aware that I may not be delivering the same quality education, and I am letting my students down, and not meeting their new needs. And their needs change with their own levels of sophistication. Am I overloading my students with all this new stuff?
**Teaching Focus**	Getting the material into the LMS. I do something in my “normal” teaching, how do I do this online? How to create webinars, podcasts, how to record PowerPoints? And PBL and CBL? What tasks are suitable, and how do I convert them? I see a host of other tools in the LMS. They look interesting, but there is no time to check. What is Zoom, anyway? How do I check all my students are there? That they stay? What does “attendance” even mean in this context? Should their cameras and mics be on? Do I just speak into the void? How can clinical teaching be converted into online scenarios? Do I just make videos with voice-over?	Quality use of the material. How do I encourage interaction? What are my colleagues doing to solve these problems? Is there a better way of doing this? The LMS is more than just a PowerPoint distribution site, so there is interesting experimentation. I found some useful tools. How do I improve these tasks and classes? Small-group work is good, but it is such hard work. Flipped classroom? Mmm. And, for the students, is it really good to have multiple devices, windows and browsers open, and all these simultaneous activities?	How has my teaching changed? How will this impact my face-to-face teaching? How is this to be sustained? Reviewing of my earlier online courses and materials; embarrassment at earlier work, and attempt to improve. How long will those courses be available to me? To my students? Should I backup everything? Refining and meeting standards. What standards are there? Local, national, international? Which is the best? What is the best practice?
**Research, Reading and Writing Focus**	Research? In all of this, you still expect me to keep up with my research? Basic, “How To” articles, Twelve Tips. This is what we did, and what we learned from it. We will do it better next time, if there is a next time.	What works best in different situations? Students needs and expectations are changing: how to cope with this? Where can I find suggestions from experienced teachers: not “you can do this,” but rather, “I actually do this.”	Using data analytics for long-term projections. All these data - is there a role for AI in all of this? How are we supposed to meet international standards? How do we know what is effective, or are we all just groping in the dark?
**Computer usage**	Standard computers, both my own and institutional, basic, free, new software, learning about how to record, edit, upload. Using known software in ways not previously used. (And recording takes so much extra time and effort, and working from home has its own complexities).	More advanced software, and using the more advanced features of the basic software, using non-standard apps and social media. Using software like a pro (I think); demanding more from the software than before. Beginning to hit institutional and other limits; finding work-arounds. Learning about cloud-computing.	More professional editing tools; collaborative production and editing. Developing more tailored systems. But students have so many different devices and they don’t all work equally. Looking for extra plugins, extra enhancements, specialised software. Hope to experiment with AI. Technological limits are now impacting on my teaching. I need a new computer. I’d be lost without cloud-computing. Work-arounds are too complex and time-consuming. How can I create Khan-academy-level materials?
**Theoretical knowledge and self-growth**	New terminology: synchronous versus asynchronous, encryption, hybrid, HyFlex, online breakout rooms. Feeling swamped in the jargon soup - it’s easier just to go with the practical side of things. (And if I hear “new normal” once more, I will scream.)	Is there any theoretical background or justification for what I am doing? Where do I find it? Am I really doing the right stuff? Am I really that bad a teacher? Is it all just hit and miss? When it works, I don’t even know why, but I repeat it and hope that it works again.	There’s an entire world of theoretical approaches to online learning. It’s not like face-to-face teaching, and it wasn’t just me. Why did I not know about this stuff before? Peer-observation? What?
**Assessment**	How to create online quizzes, all the settings, how to prevent cheating, should we use proctoring software or not, and how? How can clinical assessment be converted into online scenarios?	What other assessment tools exist? This basic stuff doesn’t do the job. Video assessment? Audio and video feedback? How to do OSCEs and OSPEs online? Open-book exams? Case-based scenarios with multiple questions attached? Item banks and randomisation?	Re-evaluation of our assessment assumptions. What about those analytics? Could they be used for assessment? What are the ethics of proctoring, especially if it requires a 360-degree view of a student’s bedroom? Is that even legal? And clinical skills assessment? What are we assessing? How far to compromise? This is a logistic nightmare. Does everyone just lurch from exam to exam?
**Institutional Support**	Encouragement mainly; support services are mostly overwhelmed; I am on my own, and rely on the old champions whose quirky tech knowledge now appears crucial (They quickly become my “go-to” every time I have a problem, but I feel guilty.) Official support may run some workshops, so I need to attend workshops and seminars, but they’re so long-winded, and I don’t have the time. Why don’t they just make videos? I wish someone would settle on a technical standard, because I cannot keep jumping around all the time.	Slightly improved support, links to other sites. I am becoming more self-reliant; forming groups; less reliant on support for the basics, making extra demands, but still relying on the champions. I also have to face 150 students complaining about the quality of the work. But I have great sympathy for the support staff attempts - I see some of them working far beyond the expected, with little acknowledgement. There may be financial support, but the institution is struggling to meet the demand and the new requirements.	I now require more sophisticated support from the Institution; online is not merely a copy of face-to-face, and the administration does not seem to understand that. Colleagues sometimes ask *me* for tech support; official support does not match my needs, leading to frustration. They still want to teach me how to build a quiz; I’m beyond that. I don’t need another tech workshop; I want input from educators. Are they even aware of international standards? I’m responsible for my course, but they call the shots. They ask: “Why do you want to do that?”, and I answer “Because I’m trying to be the best possible teacher.” But that doesn’t seem to mean anything to them. They seem to block all my attempts at innovation with the words, “Budget” or “Security” or “No-one else has asked for that.” And then I see them struggling and feel guilty.The Centre is not holding...

## And it progresses into the future

And all the time, the pressure builds and deepens: deliver the same quality of education that you did before this started. Simultaneously, there are anecdotal reports of institutions’ wishing to keep the class recordings, using them to replace teachers. (From social media, we learn that recordings are already being used to teach classes after the teachers have died ( Ansuini, 2021)).

**Figure 1:**
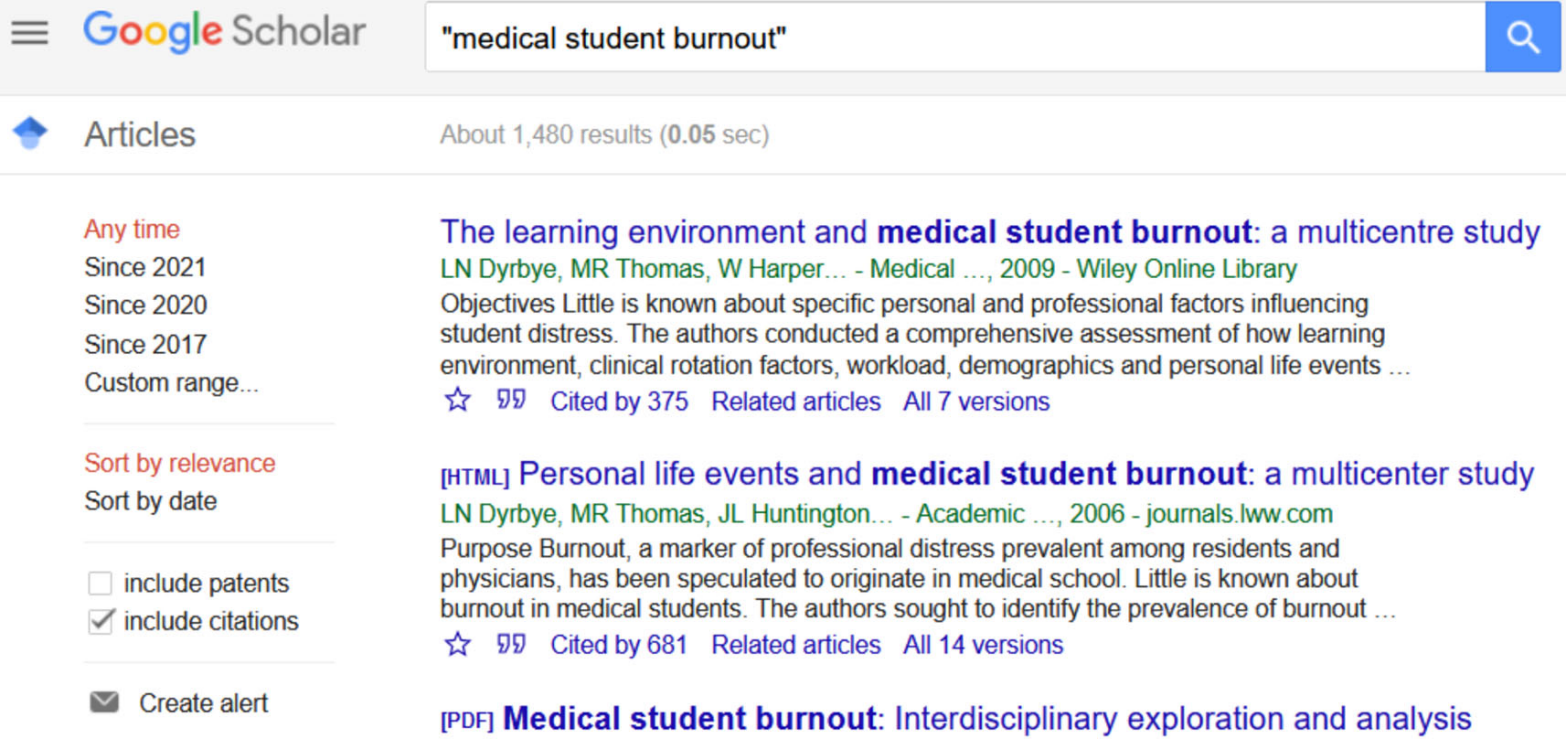
Results of a Google Scholar search on the phrase “medical student burnout” (12/02/2021)

**Figure 2:**
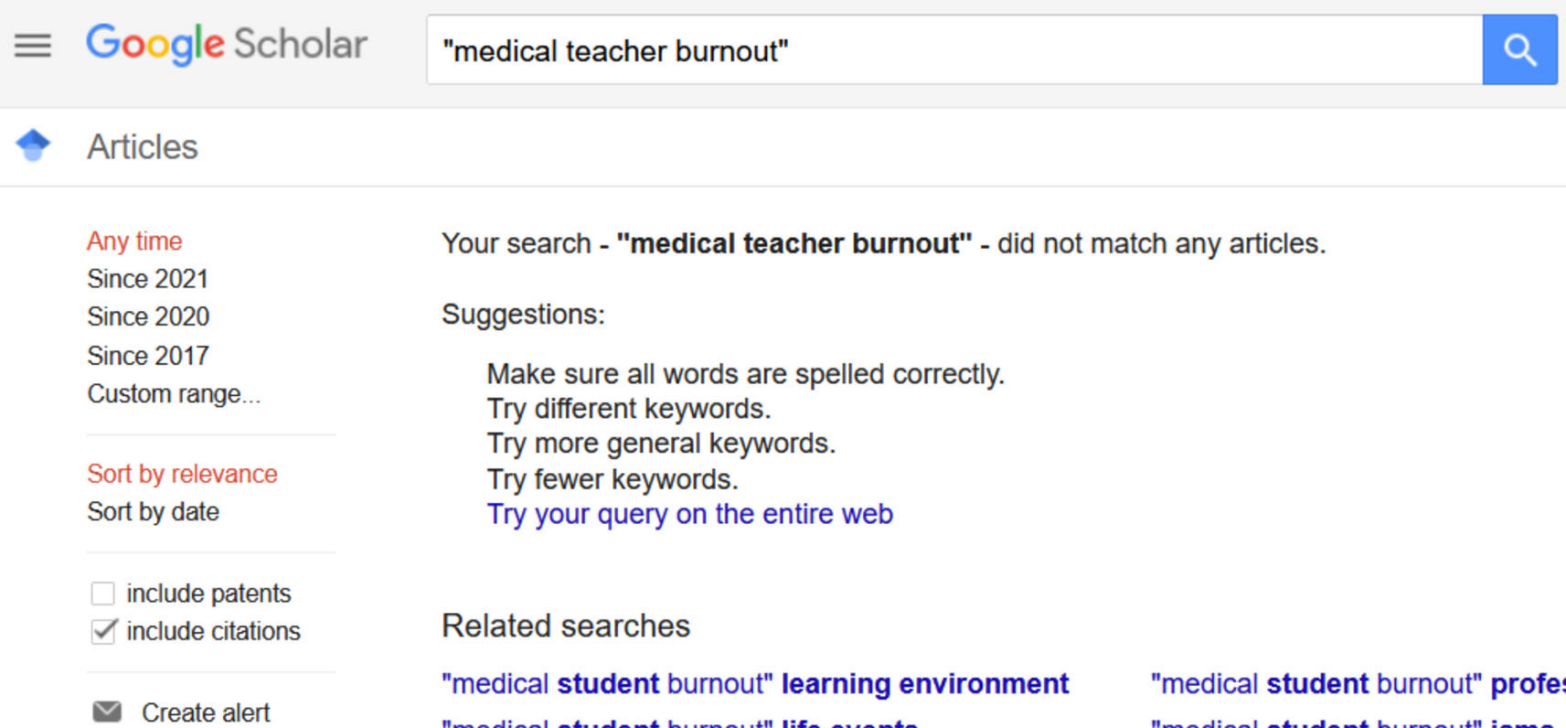
Results of a Google Scholar search on the phrase “medical teacher burnout” (12/02/2021)

Medical education literature has many studies of medical students’, residents’ and physicians’ burnout; not so much on medical teachers. (A February 2021 Google Scholar search on “medical student burnout” returned 1,480 results; a search on “medical teacher burnout” returned **Zero** results). See Figure 1 and Figure 2.

While the few scattered texts that exist are useful, they generally do not deal with a world-wide pandemic; that is not their fault, but it does mean we are working with unknowns. And the unknowns need to be addressed.

Because the unknowns will vary widely from institution to institution, the first action required is for institutions to ask (though short surveys, focus groups, or whichever process best suits all), what the specific issues are, and then obtain suggestions for responding. After that, it will be imperative that there is a swift, adequate and appropriate response; beginning immediately with smaller issues, and then the development of longer-term strategies. In addition, if the facilities exist, formal and informal counselling may be required.

The unknowns will need to be addressed now and in the future, not only because (we hope) higher education has been altered forever, but also because there will be more pandemics, and we do not wish to be caught unprepared for the next one. There is already discussion about medical education technology use after COVID-19 ( Goh and Sandars, 2020); we need to ensure that we have a secure base on which to work.

## Conclusion

In the film, *They Shoot Horses, Don’t They?* (spoiler alert), the main characters remove themselves from the competition when they realise that the rewards do not match their expectations, and pursuance is futile. Other characters continue; they may or may not know that the rewards are trivial; either way, they keep going because of their desperation and the fact that they have already invested too much into the process, even though very little of their activities match their ideals.

Most were not aware that this would be such a long-term exercise.

Many medical teachers are reaching the points of exhaustion and dejection. Medical Education institutions need to recognise what is happening to their medical teachers, and take immediate action before it is too late. Otherwise, one way or the other, medical education will suffer. Yowza! Yowza! Yowza!

## Take Home Messages

• Medical teachers are struggling to cope with the demands brought on by the COVID-19 pandemic, and many are reaching exhaustion.
• The typical stages through which the medical teachers progress can be viewed through several characteristics.
• Medical schools need to take note, anticipate, and take steps to alleviate the situation in order to prevent medical teacher burnout and the damage to medical education.

## Notes On Contributors

Ken Masters (PhD, FDE) is Associate Professor of Medical Informatics in the Department of Medical Education and Informatics, Sultan Qaboos University, Oman. He has been involved in e-learning, education and medical education for some two decades, and serves on AMEE’s Technology Enhanced Learning (TEL) Committee. ORCID: https://orcid.org/0000-0003-3425-5020.

## Declarations

The author is an Associate Editor of MedEdPublish. This paper has been submitted through the standard MedEdPublish process.
